# Root Parameters Show How Management Alters Resource Distribution and Soil Quality in Conventional and Low-Input Cropping Systems in Central Iowa

**DOI:** 10.1371/journal.pone.0164209

**Published:** 2016-10-28

**Authors:** Patricia A. Lazicki, Matt Liebman, Michelle M. Wander

**Affiliations:** 1 Department of Natural Resources and Environmental Sciences, University of Illinois at Urbana-Champaign, Urbana, Illinois, United States of America; 2 Department of Agronomy, Iowa State University, Ames, Iowa, United States of America; USDA Agricultural Research Service, UNITED STATES

## Abstract

Plant-soil relations may explain why low-external input (LEI) diversified cropping systems are more efficient than their conventional counterparts. This work sought to identify links between management practices, soil quality changes, and root responses in a long-term cropping systems experiment in Iowa where grain yields of 3-year and 4-year LEI rotations have matched or exceeded yield achieved by a 2-year maize (*Zea mays L*.) and soybean (*Glycine max L*.) rotation. The 2-year system was conventionally managed and chisel-ploughed, whereas the 3-year and 4-year systems received plant residues and animal manures and were periodically moldboard ploughed. We expected changes in soil quality to be driven by organic matter inputs, and root growth to reflect spatial and temporal fluctuations in soil quality resulting from those additions. We constructed a carbon budget and measured soil quality indicators (SQIs) and rooting characteristics using samples taken from two depths of all crop-phases of each rotation system on multiple dates. Stocks of particulate organic matter carbon (POM-C) and potentially mineralizable nitrogen (PMN) were greater and more evenly distributed in the LEI than conventional systems. Organic C inputs, which were 58% and 36% greater in the 3-year rotation than in the 4-year and 2-year rotations, respectively, did not account for differences in SQI abundance or distribution. Surprisingly, SQIs did not vary with crop-phase or date. All biochemical SQIs were more stratified (*p*<0.001) in the conventionally-managed soils. While POM-C and PMN in the top 10 cm were similar in all three systems, stocks in the 10–20 cm depth of the conventional system were less than half the size of those found in the LEI systems. This distribution was mirrored by maize root length density, which was also concentrated in the top 10 cm of the conventionally managed plots and evenly distributed between depths in the LEI systems. The plow-down of organic amendments and manures established meaningful differences in SQIs and extended the rhizosphere of the LEI systems. Resulting efficiencies observed in the LEI grain crops indicate that resource distribution as well as abundance is an important component of soil function that helps explain how LEI systems can maintain similar or greater yields with fewer inputs than achieved by their conventional counterparts.

## Introduction

### 1.1 Low external input system effect on soil quality

Farmers are under pressure to increase input use efficiency [1]. Low external input (LEI) diversified cropping systems aim to improve efficiency by manipulating natural processes to partly supply crop nutrients and reduce weed competition, instead of entirely relying on chemical inputs [2]. Many studies have documented soil quality changes under LEI systems compared with conventional systems, i.e.[3–7], and many have reported their similar agronomic performance, i.e. [2,8–10]. However, few studies have linked soil quality to crop response or have provided insight into how LEI component practices interact to drive changes in soil quality or plant response. Interactions between component practices and plant-soil response will not be the same for every crop, climate or soil type [9]. This study sought to explore how increased efficiency may be achieved for maize (*Zea mays L*.) and soybean (*Glycine max L*.) grown on an agriculturally important soil in the Midwest region of the United States.

Previous work has identified several ways in which LEI management practices can improve biochemical and physical soil properties, and thereby increase nutrient use efficiency (NUE), through the use of green and animal manures [11,12]. These organic matter additions, especially the inclusion of crops with large and potentially recalcitrant root systems, may result in a buildup of total and labile soil organic C (SOC), improving water and nutrient retention, soil structure and nutrient provision [13–17]. The extensive root systems associated with soil-improving crops such as oats or alfalfa can also enhance soil structure and the nutrient cycling environment by aerating and loosening the soil [18], fostering aggregation [17] and supporting microbial activity [19]. However, the effects of particular management practices are complex. While it’s often assumed diversified systems add more C than conventional maize-soybean systems, this may not be the case for systems that include perennial forages [5]. Additionally, the extent to which C inputs build organic matter varies with climate and soil mineralogy; thus, increased additions do not necessarily increase SOC, particularly in potentially C-saturated soils [20,21] or where high levels of available N induce organic matter priming [22].

Further, the fate of organic matter additions is influenced by disturbance and placement regimes. Tillage, which is normally used in LEI systems to incorporate manures and control weeds, may also reduce expected soil quality enhancements in the surface soil [23]. Tillage can break soil aggregates and deplete SOC [24,25]. The extent to which tillage damages soil quality depends on its frequency in the crop rotation [26]. However, the effect of tillage intensity is complex in the case of LEI systems, where tillage frequency also indicates frequency of manure and residue incorporation [7]. Tillage also changes the stratification of many soil quality indicators (SQIs). Less-tilled systems tend to accumulate SOC and concentrate its associated benefits in the surface soil, while systems in which C is incorporated are more likely to accumulate SOC deeper in the soil [27]. Whether incorporating C results in overall improved soil quality compared with less-tilled systems in which residues remain on the surface is a function of climate, soil texture, and organic amendment quality and quantity [27,28].

### 1.2 Root response to soil quality changes

Multiple studies have demonstrated that cereal crops grown in LEI or organic systems can produce yields comparable to, or greater than, those achieved in simpler annual crop systems receiving greater chemical inputs [4,8,10,29]. Maize and soybean rooting characteristics may provide insight into how soil quality influences crop response if we assume that root length density (RLD) is positively related to desirable rooting environment—i.e., aeration, nutrition and reduced soil strength [11,30–32]. Maize root proliferation has been observed in zones of higher nutrient and water availability [31,33]. Conversely, in compacted and poorly structured soil, roots encounter mechanical impedance that reduces their growth, limiting shoot growth and yield [11, 30]. If root growth were limited by soil strength, bulk density (BD) would be expected to inversely correlate with RLD and positively correlate with root average diameter (RAD) [34]. The effect of soil strength on root distribution has been observed to be stronger for grass species than for tap-rooted species [34]. Lack of oxygen may also limit root growth, particularly in fine-textured soils [35]. If root growth is limited by low oxygen, RLDs could be expected to be low in areas where the percentage of water-filled pore space (%WFPS) exceeds 60% [36].

### 1.3 Study objectives

Our objective was to document the effect of management factors on short- and long-term changes in SQIs and associated maize and soybean rooting characteristics at the Marsden Farm cropping systems experiment in Iowa, where LEI soybean yields have equaled or surpassed those of the 2-year conventional system since 2004, as have LEI maize yields since 2005 (Table 1). We expected that: 1) LEI systems would have better soil quality than their conventional counterpart, 2) temporal fluctuations in SQIs would be associated with the intensity and timing of management factors including tillage, organic matter inputs, and type of crop, and that 3) RLD of major crops (maize and to a lesser extent soybean) would be greater where soil quality was enhanced.

**Table 1:** Summary of cropping system management^a^^,^^b^, inputs^c^ and analysis^d^ 2002–2008.

System	Crop	Tillage	Inorganic N	Compost N	Avg grain yield	LEI>CONV
			(kg N ha^-1^)	(kg N ha^-1^)	(Mg ha^-1^)	
2	Maize	Spring field cultivation	143		12.3	
	Soybean	Chisel previous fall/ spring field cultivation	3		3.38	
3	Maize	Moldboard plow previous fall/ spring field cultivation^b^	53.5	128.2	12.57	2005–2006
	Soybean	Chisel previous fall/ spring field cultivation	3		3.56	2004–2007
	Oat/red clover	Zero till or spring disking	17			
4	Maize	Moldboard plow previous fall/ spring field cultivation	40.8	128.2	12.71	2005–2007
	Soybean	Chisel previous fall/ spring field cultivation	3		3.54	2004–2007
	Oat/alfalfa	Zero till or spring disking	17			
	Alfalfa	None	3			

Experiment was laid out in a randomized complete block design with four replicates, with each crop phase in each system present in each block in each year. Values represent annual averages, 2002–2008. Full experimental design and historic yield data presented in [2] and (Cruse et al., 2010).

^a^ Depth of chisel plowing approximately 15 cm

^b^ Depth of moldboard plowing approximately 20 cm

^c^ Inorganic N was applied as preplant urea and sidedressed urea ammonium nitrate.

^d^ LEI>CONV represents the years (2002–2008) in which LEI yields significantly (p<0.05) exceeded conventional system yields.

## Materials and Methods

### 2.1 Site description and management

The Marsden Farm Cropping Systems Experiment is located in Boone County, Iowa (42°01’ N; 93°47’ W; 333m above sea level). The trial consists of a conventional two-year rotation (maize and soybean; 2YR system), and two LEI systems: maize-soybean-oats (*Avena sativa* L.) intersown with red clover (*Trifolium pratense* L.; 3YR system) and maize-soybean-oats intersown with alfalfa (*Medicago sativa* L.), which then goes on to a second year (4YR system) laid out in a randomized complete block design with all crop phases in all systems replicated four times.

Soils vary across the experimental site and are predominantly Clarion loam (fine loamy, mixed, superactive, mesic, Typic Hapludolls, 2%–5% slope), Nicollet loam (fine-loamy, mixed, superactive, mesic, Aquic Hapludolls, 1–3% slope) and Webster silty clay loam (fine-loamy, mixed, superactive, mesic, Typic Endoaquolls, 0–2% slope), with smaller areas of Harps loam (fine-loamy, mixed, superactive, mesic Typic Calciaquolls, 0–2% slope), and Canisteo silty clay loam (fine-loamy, mixed, superactive, calcareous, mesic Typic Endoaquolls, 0–2% slope) [2]. Prior to the start of the experiment in 2002, the site had a mean pH of 6.8 and SOC concentration of 29.65 g kg^-1^ in the top 20 cm [2]. In 2009, soils contained on average 350 g kg^-1^ sand, 384 g kg^-1^ silt and 265 g kg^-1^ clay. Plots were 9 m by 85 m with 16 m buffer strips between blocks. Plots were split in two as part of an unrelated experiment, and samples were taken from the halves that had similar maize and soybean genotypes and received similar herbicide regimes. System tillage, nutrition and historical yields are outlined in Table 1. More inorganic N is added to the 2YR system; however, the two LEI systems receive organic N as incorporated green manures and composted cattle manure (Table 1). Assuming typical N fixation rates, the 3-year and 4-year rotations receive approximately 20–35% of their N inputs from biological fixation. Tillage consists of fall chisel plowing (depth = 15 cm) and spring cultivation in the 2YR system, while the two LEI systems undergo inversion tillage (depth = 20 cm) prior to the maize year for manure incorporation and weed control. The 3YR system receives both manure incorporation and tillage with greater frequency than the 4YR system. Full management details are reported in [2] and [37].

### 2.2 Soil sampling and analysis

To evaluate system level differences, we sampled in May 2009 and 2010 around the time of planting and after any spring soil disturbance. We sought to correlate temporal fluctuations in SQIs to management practices (tillage, inputs and/or crop phase) by taking samples three times in 2009 (May, July around the time of maize and soybean maximum root development and just before the oat harvest, and in late September around maize and soybean harvest time). At all sampling dates, we obtained cores from all crop phases at 0–10 and 10–20 cm depths. These depths were chosen to reflect the depth of moldboard plowing, and to allow comparisons with baseline samples that were taken at the site in 2002. Composite samples were formed from four large diameter (5.08 cm) soil cores. To quantify management effects on soil physical characteristics we measured BD, percent water-stable aggregates (%WSA), and %WFPS. To assay biochemical characteristics we measured SOC, particulate organic matter C (POM-C), potentially mineralizable N (PMN) and the fluorescein diacetate hydrolysis rate (FDA, a measure of heterotrophic microbial activity). These indicators were chosen because they are sensitive to management and positively correlated with C and N mineralization and crop yields (i.e. [7,36,38–40].

Soil BD was determined using the core method. All SQI values were corrected for BD and reported on a volume basis. Percent WFPS was calculated after Linn and Doran [36] as:

 %WFPS=(F)*(D)/(1−D/2.65) (1)

where F is the soil water content and D is BD. The remaining soil was sieved to 8 mm and air-dried. The 8 mm fraction was used to measure %WSA using a standard wet-sieving method [41]. The remaining soil was mechanically crushed to pass through a 2 mm sieve for use in soil texture, total SOC, POM-C, FDA and PMN determination. Soil texture was analyzed on soils from the summer of 2009 only, using the standard hydrometer method [42]. In fall 2009 only, soils were finely ground and analyzed for total C and N by dry combustion. As the soil was found to contain carbonates, SOC was obtained by subtracting inorganic C values determined for each sample as described by [43]. Soil POM (the organic matter fraction between 53 μm and 1000 μm) was extracted as described by [40]. The POM-C fraction was determined by dry combustion. The >53 μm fraction in which POM-C was measured contained carbonates, and thus we developed appropriate correction factors by removing carbonates via fumigation with HCl as described by [44]. These correction factors were based on difference where the percent of C in carbonates was assumed to equal C in the uncorrected minus the corrected samples, and were developed using soils collected in spring 2010 and applied to other dates. Soil PMN was determined with an anaerobic incubation as described by [40]. Microbial enzymatic activity was measured using the FDA hydrolysis method, modified from that proposed by [39] as described by [40]. Briefly, soil samples are reacted with fluorescein diacetate (FDA), which is hydrolyzed by a broad spectrum of C-cycle enzymes to yield fluorescein, which can be analyzed on a spectrophotometer. The reaction is stopped by acetone and samples are extracted and analyzed colorimetrically at 650 nm. The FDA hydrolysis rate is measured as μg FDA hydrolyzed cm^-3^ soil min^-1^.

### 2.3 Plant sampling and analysis

To quantify crop C input to the soil and to evaluate relative crop growth, harvested portions and aboveground stover were obtained from all crops in 2009. In fall directly before soybean harvest and after maize had dried down, maize grain and stover were collected from four representative plants near the center of the plot, and soybean grain and stalks, pods and leaves were sampled from two 0.58 m^2^ sample areas near the north and south ends of all plots. In October before plow-down, aboveground biomass samples of alfalfa and red clover were taken from four 0.25 m^2^ sample areas. Plant samples from each plot were composited, dried at 40°C and ground before total C and N were determined by dry combustion. Grain removed from the maize and soybean crops was dried and weighed. Values from 2008 for grain and manure C and N concentrations and mass of manure additions were obtained from the Marsden field staff.

To evaluate rooting characteristics we measured RLD, RAD and C to N ratio on the roots of all crops in all systems. Root cores (5.08 cm diameter) were obtained from 0–10 and 10–20 cm depths adjacent to soil sampling locations, and composited. Samples from soil under oat were taken in late June just before the oat harvest. For all other crops, cores were taken in late July. Root cores were also taken at the fall 2009 soil sampling to assess root C input for the C budget. Root cores were put on ice after collection and kept at 4°C until analysis within a month from the time of collection. Roots were elutriated, cleaned and stored in 50% ethanol at 4°C. The RLD and RAD were measured on an Epson 1680 scanner (Epson America Inc., Long Beach, CA, USA) utilizing WinRhizo root scanning software (WinRhizo, Regent Instruments, Québec, Canada). After scanning, root samples were dried, weighed, ground and analyzed for C and N by dry combustion. Root exudates were estimated as 0.65 times the measured summer root C, after [45]. This method of root sampling, while sufficient for comparing average root length density and diameter between crops and systems, considerably underestimates root mass and C as it does not capture crowns or taproots. Extrapolation to field scale estimates will therefore be lower than actual values, particularly for tap-rooted plants.

Total C inputs to crop X were calculated as:

$$C_{X}=\left(A_{X}\right)+\;\left(B_{X}\right)+\left(M\right)\;+\;\left(E_{X}\right)$$ (2)

and average annual C inputs to each system were calculated as:

$$\bar{C}=\sum_{i=1}^{n}C_{X}*n^{-1}$$ (3)

where C_X_ is the total C input associated with crop X, A_X_ is the aboveground residue C from crop X, B_X_ is the belowground residue C from crop X, M is the composted manure added the previous fall, E_X_ is the estimated exudate C from the roots of crop X, $\bar{C}$ is the average annual C input to a system and n is the number of crop-years in the rotation.

To explore the differential effect of systems on depth distribution for each soil and root variable we calculated a stratification ratio S between the depths for each plot *i* as

$$\;S_{i}=\frac{I_{10}}{I_{20}}$$ (4)

where *I*_*10*_ is equal to the value of indicator *I* at the 0–10 cm depth and *I*_*20*_ is equal to the value of indicator *I* at the 10–20 cm depth.

### 2.4 Statistical analyses

Three-factor randomized complete block ANOVAs were performed using PROC MIXED in SAS (SAS Institute, Cary, NC) to detect treatment (crop phase), soil depth (0–10 cm; 10–20 cm) and sampling date main effects and interactions for POM-C, PMN, FDA, BD and %WFPS. Two-way ANOVAS were used for SOC and system C inputs measured in fall of 2009, rooting characteristics measured during the summer of 2009, and for SQI stratification ratios. We regarded each crop within each system as a class variable crop(system) and so considered nine classes (2Maize, 3Maize, 4Maize, 2Soy, 3Soy, 4Soy, 3Oat/legume, 4Oat/Legume, 4Alfalfa). To test the overall effect of LEI management on soil quality, we assessed the aggregate effects of management on SQI using data collected in spring of 2009 and 2010. Sampling in two years helped address challenges presented by spatial variability by ensuring that observed system differences in SQIs were not due to interactions between a particular year's weather, which was slightly cooler and wetter than normal in 2009 and extremely wet and warm during summer 2010, and random differences in soil quality that exist within plots. To examine short-term responses to management events like tillage or the presence of a particular crop, main effects and their interactions with time over the 2009 growing season were used to determine whether the timing, placement and intensity of management affected SQIs and RLD.

Comparisons between individual systems and between LEI vs. conventional systems were made with preplanned orthogonal contrasts using a stepdown Bonferroni adjustment with an alpha of 0.05. Even though blocks were included as a factor, they did not adequately remove spatial variability associated with soil texture. Concentration of clay particles (<2 μm) was used as a covariate when covariate interactions were significant at p<0.05 to remove spatial effects not effectively removed by blocks. Class variable interactions were dropped if they were insignificant at *p*>0.35. The square root transformation was performed on the POM-C, PMN, FDA and root length variables, and the natural log transformation on the stratification ratios for POM-C, PMN and FDA and on crop C inputs. Reported values are back-transformed LSMEANS and the standard deviation of the untransformed data. Differences in means were assessed using Tukey’s test, and were considered significant at p<0.05 and marginally significant at p<0.10. To evaluate the relationship between bulk density and root growth we regressed RLD and RAD against summer BD for each crop species and for the data as a whole using PROC REG in SAS. All assumptions of normality were met.

## Results

### 3.1 Questions 1 and 2

#### 3.1.1 Mass, placement and types of organic matter introduced

Average annual C inputs to both the 3YR and 2YR systems were significantly greater than inputs to the 4YR system. On average, the 3YR and 2YR systems contributed 58% and 36% more C than the 4YR system, respectively (Table 2). Carbon inputs varied significantly among cropping phases (Table 2); with average C inputs in the maize year > soybean> oat/legume> 2nd year alfalfa. The average C input in the 4YR oat/alfalfa year is lower than that of the 3YR oat/ red clover year due to biomass harvest, and the aboveground biomass C of the red clover incorporated as green manure in the 3YR system was much larger than biomass returned by the alfalfa in the 4YR system (Table 2). Due to the absence of manure and cover additions, below ground additions accounted for only 9% of C inputs to the 2YR system. In comparison, 28% of C additions to the 3YR and 38% of additions to the 4YR system were added below ground as roots, exudates or incorporated as compost and green manures (Table 2). While our sampling method underestimated root-derived C inputs, this underestimation is probably greatest for the 3YR and 4YR systems which contained a larger proportion of tap-rooted crops. Direct N additions were similar in the 2YR and 3YR systems and lower in the 4YR system (Table 1; 2YR 71, 3YR 67, and 4YR 42 kg N^-1^ ha^-1^ yr^-1^); however, based on typical assumptions for legume fixation rates, the average amount of N introduced to the systems annually (2YR: 73, 3YR: 84, 4YR: 67 kg ha^-1^ yr^-1^), look more similar.

**Table 2:** Estimated average of yearly carbon (C) inputs^a^^,^^b^^,^^c^ to each crop phase (g C m^-2^) and analysis^d^^,^^e^^,^^f^^,^^g^.

Crop phase	System	Aboveground biomass C		Root biomass C		Est. root exudates		Manure		Total C inputs	
**Average of all crop years**	2	293.1 (93.1)	a	13.15 (3.37)	a	12.56 (4.14)	A			319.2 (97.2)	a
	3	304.9 (97.9)	a	11.18 (2.59)	a	9.96 (3.25)	A	46.1 (68.0)	a	372.6 (166.6)	a
	4	175.8 (178.6)	a	12.56 (6.80)	a	11.89 (4.47)	A	34.6 (61.8)	b	235.6 (231.2)	b
**Corn**	2	374.6 (38.8)	a*	14.7 (3.53)	a	14.72 (3.71)	A			404.5 (40.3)	b
	3	428 (50.2)	a*	12.43 (3.01)	a	13.24 (3.09)	A	138.2 (0.0)		592.8 (48.9)	a
	4	413.5 (51.8)	a	12.78 (4.74)	a	12.00 (6.68)	A	138.2 (0.0)		577.6 (57.6)	a
	**Mean**	**405.4 (49.0)**	**A**	**13.30 (3.61)**	**AB**	**13.32 (4.46)**	**A**	**92.2 (68.1)**		**525.0 (99.9)**	**A**
**Soy**	2	211.5 (32.9)	b	11.59 (2.67)	a	10.39 (3.69)	A			233.9 (33.8)	b*
	3	258.4 (28.4)	a	10.30 (3.43)	a	8.85 (2.11)	A			277.8 (31.8)	ab*
	4	260.4 (22.2)	a	12.94 (2.56)	a	10.18 (4.14)	A			283.8 (22.0)	a
	**Mean**	**243.4 (34.4)**	**B**	**11.61 (2.83)**	**AB**	**9.81 (3.18)**	**A**			**265.2 (35.2)**	**B**
**Oat/legume**	3	228.5 (24.6)	a	10.80 (0.74)	a	7.80 (1.60)	A			247.2 (23.5)	a
	4	0.0 (0.0)	b	6.31 (3.49)	a	12.78 (2.81)	A			19.5 (3.4)	b
	**Mean**	**114.3 (123.7)**	**C**	**8.56 (3.11)**	**B**	**10.29 (3.40)**	**A**			**133.4 (123.0)**	**C**
**Alfalfa**	4	29.4 (4.5)		18.20 (9.18)		12.61 (4.95)	A			61.6 (7.2)	
	**Mean**	**29.4 (4.5)**	**D**	**18.20 (9.18)**	**A**	**12.61 (4.95)**	**A**			**97.5 (7.2)**	**C**

^a^ C from stover measured in fall 2009

^b^ C measured in roots collected in fall 2009.

^c^ Sum of aboveground and root biomass C from the previous crop, manure C incorporated to the crop, if any, and root exudate C calculated after [45] as 0.65*summer root C

^d^ Standard deviations are in parenthesis.

^e^ Different lowercase letters represent differences between systems in a specified crop that were significant at p<0.05

^f^ Asterisks * represent differences between systems in a specified crop that were significant at p<0.10.

^g^ Different uppercase letters represent differences that were significant at p<0.05 between values for each crop averaged over all rotations and both depths.

#### 3.1.2 Farming practices, farming systems and biochemical indicators of soil quality

The spring data from 2009–2010 shows that overall, LEI systems maintained significantly higher POM-C and PMN concentrations than the 2YR system (*p* = 0.004 and *p* = 0.01, respectively). Soil POM-C concentrations were similar in the 3YR and 4YR systems, and both were significantly greater than concentrations in the 2YR system (Tables 3 and 4). There were significant differences between the 2YR and 3YR systems’ PMN concentrations (*p* = 0.01) and marginally significant (*p* = 0.08) differences between concentrations in the 2YR and 4YR systems. The FDA in the top 20 cm did not differ among systems (Table 4). Soil POM-C, FDS, WFPS and WSA differed between years (S1 Table). However, crop(system) interactions with year were not significant (Table 3).

**Table 3:** Analysis of variance of soil properties (p-values) for soils collected in Spring of 2009 and 2010^a^^,^^b^^,^^c^.

Source	SOC	POM-C	PMN	FDA	BD	WFPS	WSA	RLD
**Crop(system)**	0.6869	0.0298	0.0894	0.8815	0.0608	<.0001	0.4608	0.1063
**Depth**	<.0001	<.0001	<.0001	<.0001	<.0001	<.0001	<.0001	<.0001
Crop(system)*Depth	0.2028	<.0001	<.0001	<.0001	0.1442	0.0027	0.014	0.0064
**Year**	NA	0.0763	0.4862	0.0008	0.0006	<.0001	0.0281	NA
Crop(system)*Year	NA	0.0829	0.0938	0.949	D	0.5748	0.3404	NA
Depth*Year	NA	0.0393	D	0.2801	0.3829	0.4232	0.0421	NA
Crop(system)*Depth*Year	NA	0.2982	D	0.105	D	0.2399	0.1542	NA
**Clay**	<.0001	0.0595	0.0062	0.0043	<.0001	D	<.0001	0.0385
	*Stratification ratio*							
**Crop(system)**	0.2733	<.0001	0.0003	0.0003	0.1925	<.0001	0.0609	0.0137
**Year**	NA	0.0493	D	0.0755	0.5867	0.0139	0.0277	NA
Crop(system)*Year	NA	D	D	0.0431	0.0986	0.0667	0.2212	NA

SOC, Soil organic C; POM-C, particulate organic matter C; PMN, potentially mineralizable N; FDA, enzymatic activity; BD, bulk density; WFPS, water-filled pore space; WSA, percentage of water-stable macro-aggregates; RLD, Root length diameter

^a^ With the exception of SOC, which was measured on samples collected in Fall of 2009, and RLD, which was measured on soils collected in summer 2009

^b^ "NA" signifies a term not included in the model.

^c^ "D" signifies a term dropped from the model with a p-value >0.35 for class variables and >0.05 for the covariate.

**Table 4:** Soil variable means and stratification for soils collected in Spring 2009 and 2010 from 0–10 cm and 10–20 cm depths ^a^^,^^b^^,^^c^^,^^d^^,^^e^.

Depth	System	SOC		POM-C		PMN		FDA		BD		WFPS		WSA		RLD	
		(Mg ha^-1^)		(mg C cm^-3^)		(μg cm^-3^)		(μg cm^-3^ min^-1^)		(g cm^-3^)		(%)		(%)		(cm cm^-3^)	
**0-20cm**	**2** (n = 32)	58.98 (6.64)	a	1.90 (1.14)	b	31.24 (17.31)	b*	0.96 (0.50)	a	1.13 (0.11)	a	49.25 (7.56)	a	55.68 (7.50)	a	2.72 (0.82)	a
	**3** (n = 48)	56.05 (7.72)	a	2.31 (0.66)	a	40.44 (13.71)	a	0.96 (0.21)	a	1.09 (0.13)	a	46.50 (10.35)	ab	52.41 (12.13)	a	2.90 (1.01)	ab
	**4** (n = 64)	58.40 (7.69)	a	2.19 (0.66)	a	37.26 (14.01)	ab*	0.92 (0.26)	a	1.11 (0.12)	a	45.48 (9.97)	b	55.73 (7.12)	a	3.41 (0.93)	b
**0–10 cm**	**2** (n = 16)	28.34 (3.42)	a	2.91 (0.61)	a*	44.46 (12.63)	a	1.29 (0.44)	a	1.05 (0.10)	a	43.92 (6.67)	a	55.56 (6.31)	a	3.35 (0.48)	a
	**3** (n = 24)	26.35 (3.93)	a	2.50 (0.58)	a	42.05 (14.42)	a	1.00 (0.22)	b	1.00 (0.12)	a	37.83 (5.13)	b	46.66 (12.15)	a	3.23 (1.07)	a
	**4** (n = 32)	27.00 (4.62)	a	2.35 (0.67)	a*	39.62 (14.67)	a	0.96 (0.26)	b	1.04 (0.09)	a	37.80 (6.57)	b	52.32 (7.23)	a	3.80 (0.96)	a
	**Mean** (n = 72)	**27.23 (4.16)**	**B**	**2.59 (0.65)**	**A**	**42.04 (14.01)**	**A**	**1.08 (0.32)**	**A**	**1.03 (0.10)**	**B**	**39.85 (6.60)**	**B**	**51.52 (9.42)**	**B**	**3.46 (0.92)**	**A**
**10–20 cm**	**2** (n = 16)	30.97 (3.57)	a	0.89 (0.41)	b	18.02 (12.48)	b	0.62 (0.34)	b	1.22 (0.07)	a	54.59 (3.78)	a	55.81 (8.32)	a	2.09 (0.27)	b
	**3** (n = 24)	29.87 (4.80)	a	2.12 (0.72)	a	38.82 (13.15)	a	0.92 (0.21)	a	1.17 (0.09)	a	55.17 (5.72)	a	58.16 (9.38)	a	2.57 (0.84)	ab
	**4** (n = 32)	31.60 (4.54)	a	2.03 (0.62)	a	34.90 (13.13)	a	0.88 (0.25)	a	1.17 (0.11)	a	53.16 (6.23)	a	59.14 (6.69)	a	3.03 (0.72)	a
	**mean** (n = 72)	**30.81 (4.36)**	**A**	**1.68 (0.79)**	**B**	**30.58 (14.93)**	**B**	**0.81 (0.28)**	**B**	**1.18 (0.10)**	**A**	**54.30 (5.63)**	**A**	**57.70 (7.94)**	**A**	**2.56 (0.78)**	**B**
		*Stratification ratio*																	
	**2** (n = 16)	0.87 (0.09)	a	3.34 (1.96)	a	2.40 (1.29)	a	1.85 (1.09)	a	0.90 (0.10)	a	0.83 (0.12)	a	0.90 (0.08)	a*	1.71 (0.33)	a
	**3** (n = 24)	0.86 (0.12)	a	1.09 (2.19)	b	1.00 (0.45)	b	1.06 (0.26)	b	0.88 (0.08)	a	0.69 (0.08)	b	0.73 (0.18)	a*	1.40 (0.67)	ab
	**4** (n = 32)	0.86 (0.14)	a	1.18 (0.46)	b	1.14 (0.49)	b	1.08 (0.29)	b	0.90 (0.11)	a	0.72 (0.11)	b	0.86 (0.08)	a	1.30 (0.32)	b

SOC, Soil organic C; POM-C, particulate organic matter C; PMN, potentially mineralizable N; FDA, enzymatic activity; BD, bulk density; WFPS, water-filled pore space; WSA, percentage of water-stable macro-aggregates; RLD, Root length diameter. All analyses run with clay as a covariate. Overall system differences values are linear combinations of the LSMEANS of all crops within each rotation, over both dates and both depths. Rotation by depth values are linear combinations of the LSMEANS of all crops within each rotation averaged over both dates but separated by depth. System differences are obtained by contrast statements comparing means of all crops within the rotations.

^a^ With the exception of SOC, which was measured on samples collected in Fall of 2009, and RLD, which was measured on soils collected in summer 2009

^b^ Standard deviations are in parenthesis.

^c^ Different lowercase letters represent significant differences between the systems at p<0.05 or significant differences between rotations at each depth at p<0.05, using a stepdown bonferroni adjustment for estimates.

^d^ Asterisks * represent differences between systems in a specified crop that were significant at p<0.10.

^e^ Different uppercase letters represent differences that were significant at p<0.05 between values for each crop averaged over all rotations and both depths.

Variation in depth distribution was highly significant (*p*<0.0001) for all biochemical indicators (Table 3). While POM-C and PMN concentrations in the 0–10 cm depth were not significantly different among systems, soil POM-C and PMN concentrations in the 10-20cm depth LEI systems were significantly higher (232% and 204% respectively; Table 4) than those in the 2YR 10-20cm depth. In the 0–10 cm depth, FDA in the 2YR system was significantly higher than in either LEI system, but in the 10–20 cm depth both LEI systems were significantly higher than the 2YR system (Table 4). For all three biochemical SQIs the LEI systems had markedly (*p*<0.001) lower stratification ratios than the 2YR system (Table 3). Soil quality in the 2YR system was highly stratified (Table 4). This was not the case for the LEI systems, which had ratios approaching 1 for all biochemical SQIs (Table 4). We found no significant system differences in total SOC concentrations, SOC distribution or stratification ratios (Tables 3 & 4). In all systems, SOC concentrations adjusted for bulk density were higher in the 10–20 cm sampling depth because bulk density was higher (Table 4).

**Fig 1:**
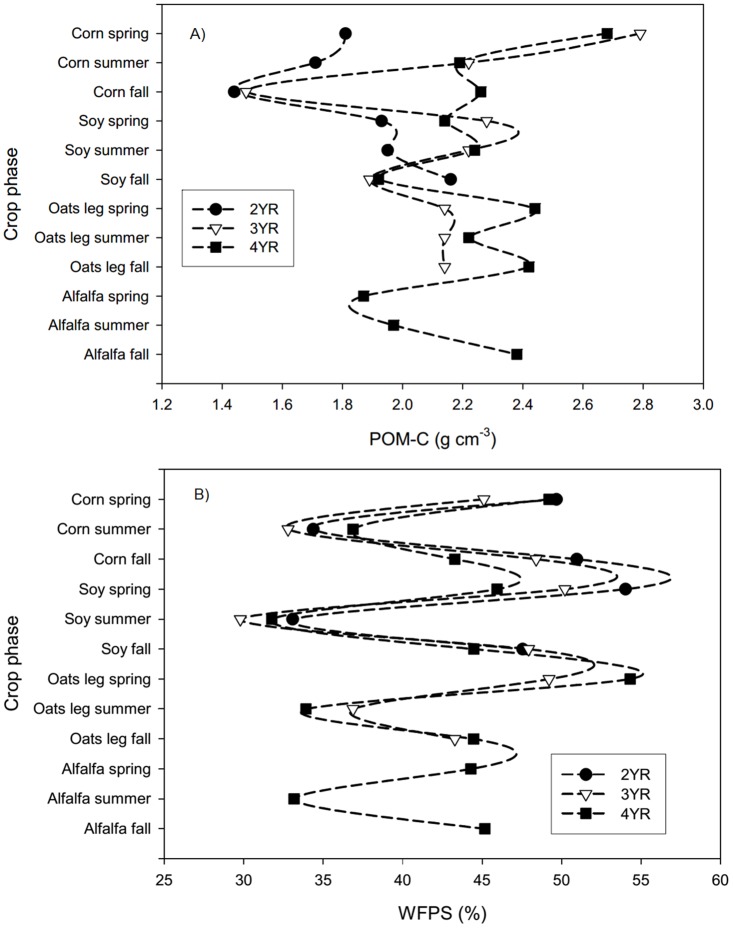
Seasonal changes in soil quality indicators in the 2009 growing season. A) Particulate organic matter C (POM-C) and B) percent water-filled pore space (%WFPS) in different crop phases of 2YR conventional and 3YR and 4YR LEI systems during spring, summer and fall of 2009.

Despite notable differences in the mass, type and distribution of C contributed by the different crop phases, POM-C, PMN and FDA remained remarkably stable within the systems during the 2009 season and over the course of the rotation as represented by measures taken in spring, summer, and fall of each crop phase (S1 Table). Contrary to our second expectation, we did not find any significant temporal differences among crops within systems in any of the biochemical SQIs, or changes directly following management events (S1 and S2 Tables). The only marginally significant temporal trend observed was a decrease (*p* = 0.008) in POM-C concentrations that occurred between spring and fall during the maize phase of the 3YR rotation, where POM-C fell from a spring high of 2.79 g cm^-3^ to a fall value of 1.48 g cm^-3^ (Fig 1A). This was the only instance where a particular practice, eg: green manure incorporated the previous fall, could be logically tied to fluctuations in labile C stocks.

#### 3.1.3 Cropping systems and physical indicators of soil quality

All physical SQIs varied within the growing season (*p*<0.0001; S1 Table) and between spring 2009 and 2010 sampling dates (Table 3). No significant crop within systems effects or interactions with date were observed for BD and %WSA, suggesting fluctuations were not driven by management. Overall, all treatments had a relatively low BD (spring average of 1.1 g cm^-3^) and high %WSA (spring average of 55%), which did not differ among crops or systems (Tables 3 & 4). However, with spring %WSA we observed that while the 0–10 cm soil was less aggregated than the 10–20 cm soil in all systems, this effect was slightly stronger (*p*<0.10) in the 3YR system than in the 2YR system (Table 4). The 4YR system was intermediate.

Soil %WFPS also varied notably with season, and interacted strongly (*p*<0.0001) with cropping system (S1 Table). In all systems, the %WFPS was high in spring and dropped in summer, and tended to rise again in fall to near spring levels. The exception to this pattern occurred during the oat/legume year in both LEI systems, during which plots were somewhat drier in fall (Fig 1B). The drying effect of the oat/legume phase was statistically significant in the 10–20 cm depth. Soils under LEI management were drier on average than those that were conventionally managed whether comparing seasonal averages for the 2009 growing season (*p* = 0.003), or springs of 2009 and 2010 (*p* = 0.01). In spring of 2009 and 2010, the 4YR system was significantly drier overall than the 2YR system; and, the 3YR system was intermediate. However, system-based spring moisture differences were restricted to the surface layer; while the 0–10 cm depth was drier in LEI systems, moisture levels were similar in the 10–20 cm depths of all systems (Table 4).

### 3.3 Question 3; yield and root response to soil quality

In 2009, maize yield in the 3YR system (12.61 Mg ha^-1^) was significantly higher than yield in the 2YR system (11.41 Mg ha^-1^), and maize yield in the 4YR system was intermediate (12.39 Mg ha^-1^). Soybean yield was higher in the 4YR system (3.45 Mg ha^-1^) than in the 2YR system (2.92 Mg ha^-1^), and soybean yield in the 3YR was intermediate (3.30 Mg ha^-1^). These differences are in line with a trend observed at these sites where LEI grain yields tend to slightly exceed conventional yields (Table 1). While soybean biomass was significantly greater in both LEI systems than in the 2YR system, differences in maize biomass were not statistically significant (Table 2).

**Fig 2:**
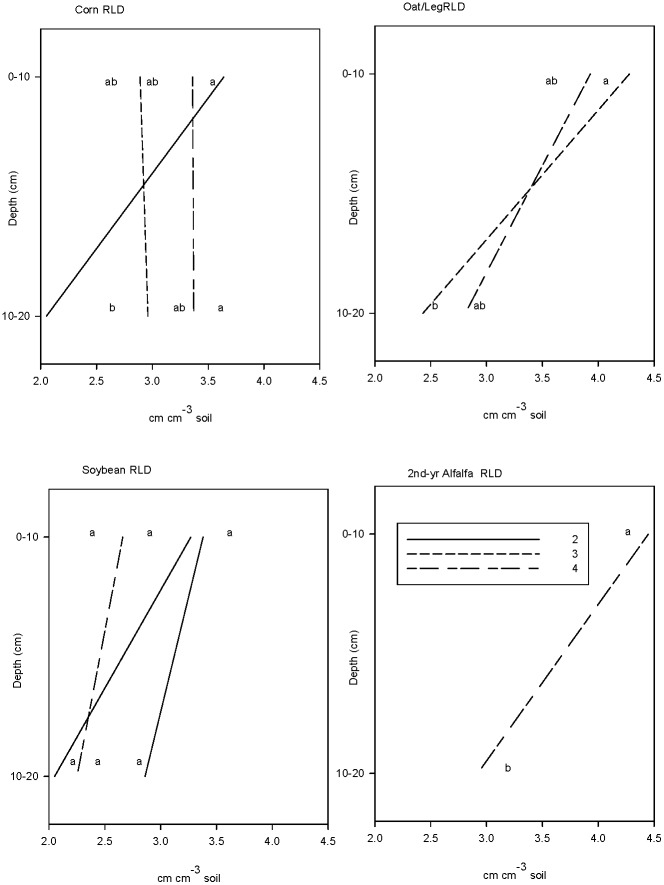
Root length density in summer of 2009. Mean RLD in 2-yr (^**___**^), 3-yr (….) and 4-yr (^**_ _ _**^) systems, determined on root cores taken at maximum root development. Different letters denote significant differences between systems and depths within crops at *p*<0.05.

Maize RLD ranked 4YR >3YR >2YR and differences between the 4YR and 2YR system were significant (Fig 2). The 2YR maize RLD was significantly more stratified than that of the LEI maize (Fig 2). While the soybean RLD tended to follow the same pattern as the maize RLD, the stratification differences were less pronounced and not statistically significant (Fig 2). There was a trend for root stratification in the LEI systems to increase with years after moldboard plowing; i.e. corn>soybean>oat>alfalfa (Fig 2). Soybean root C to N ratios were 22.8, 18.0, and 15.6 in the 2YR, 3YR and 4YR systems respectively. The difference between the 2YR and LEI soybean root C to N ratios was marginally significant (*p* = 0.06). We found no significant relationship between bulk density and RLD or RAD, either over the whole dataset or for individual crops (S3 Table).

## Discussion

### 4.1 Differences between soils under conventional and LEI cropping systems

Our results support the expectation that soil quality in the top 20 cm of the LEI systems would be improved compared with their conventional counterpart, in that POM-C and PMN were slightly increased in both LEI systems. However, the more striking difference was the uniform distribution of the biochemical SQIs observed within the top 20 cm of soil in the LEI systems; this contrasted with the highly stratified resource distribution found in the conventionally managed soil. The burial of organic materials through tillage appears to be a key contributor to increased stocks of labile organic matter, evidenced by POM-C and PMN that were found in the LEI systems. Labile and total C stocks were affected more by the placement than by the quantity of C inputs. Moldboard plowing incorporates residues deeper in the LEI systems while chisel plowing leaves residues near the surface in the 2YR system. High SOC stratification ratios are a characteristic of reduced till systems and have been considered to be indicative of improved soil quality, particularly where water erosion is of import [46]. However, this assumes a relative enhancement of surface soil quality. In the Marsden Farm soils only FDA, which is a measure of C cycle enzyme activity and hence decay rates, and %WFPS were increased in the top 10 cm of the 2YR system relative to the LEI systems. Enhanced biological activity observed in the surface soil of the 2YR system may have been associated with higher soil moisture contents and greater inputs of residues and urea made to the surface of that system [47]. Rapid mineralization of SOC, or positive priming effects, can happen when microbial activity is stimulated by the addition of easily available organic substances, and of N fertilizers which lower the C to N ratio [22]. A priming effect could help explain why SOC concentrations or stocks of biochemical SQIs might be lower than expected for a soil receiving relatively large amounts of C and less tillage than its LEI counterparts. Soil N status could also help explain the dampened response to C inputs in the labile organic matter fractions by promoting rapid decay of labile C stocks [48]. Observed PMN levels exceeded values that Ugarte and Wander [40] associated with accelerated decay in all but the subsurface soils in the 2YR system.

Our findings are consistent with Cavigelli et al. [28] who found that tilled organic grain systems which included manures or cover crops could accumulate SOC and labile C to an equal or greater extent than reduced- or no-till conventionally fertilized systems. However, unlike our study, they associated increases in SOC and labile fractions with greater organic inputs. The Marsden plots' apparent insensitivity to C inputs may have a couple explanations. The first may be depth of sampling, which was restricted to a depth of 20 cm to match baseline samples and the depth of plowing. Results may have differed had we sampled more deeply, as there is evidence that SOC can accumulate just below the depth of plowing. Future research should extend the depth of sampling to assess C sequestration. An alternative and plausible explanation for our failure to find response to C inputs would be that these soils are C saturated. This means increasing C inputs do not increase C stocks because the soil’s protective capacity, defined as the amount of C that can be physically associated with silt- and clay-sized particles and small microaggregates, has been exceeded [49,50]. Because mineral-associated SOC is more resistant to decomposition than labile fractions that are either occluded by aggregates or loose within the soil [49](Six et al., 2002), the fine fraction largely determines SOC equilibrium levels in soils. In C saturated soils, the relatively small increases in C stock size resulting from accrual within occluded fractions are difficult to detect, particularly in high SOC soils. A study done on a fine textured soil in Prairie City, Iowa by Guzman and Al-Kaisi [51] provides an example of how recovery of SOC might work in LEI systems maintained on such soils. Working on a reconstructed prairie soil, they found soils accumulated C quickly after initial conversion from agricultural use to prairie, but that SOC in the top 15 cm began to plateau at around 40 Mg C ha^-1^ after about 10 years after establishment [51]. This is lower than SOC levels found in an undisturbed prairie remnant located nearby in a slightly heavier textured soil that has maintained a C content of 55 Mg ha^-1^. The equilibrium levels achieved by the restored soil are similar to those found in the Marsden soil, which, adjusted to a 15 cm depth, average 43 Mg C ha^-1^. The idea that soils in the surface depth are C saturated in the Marsden trial is further supported by the results from incubation experiments done by Chen et al. [52], that found a higher proportion of C was mineralized from wheat residues added to Marsden Farm soils than from residues added to similarly managed Maryland soils that had lower SOC concentrations due to their mineralogy. In addition, our observed buildup of labile C without an attendant buildup of total SOC is characteristic of manured soils whose protective capacity has been exceeded [20].

### 4.2 Evidence of individual crop and management effects

Overall, specific management events (placement, timing and mass of material added through plant and fertilizer inputs) had little discernible effect on our soil quality measures. Our data did suggest that type of input and soil drying by cover crop roots both affected observed SQI dynamics. One of our most surprising results was that biochemical SQIs were similarly elevated in both LEI systems despite the 3YR rotation’s greater and more frequent OM inputs. The rapid pulse and decline of POM-C observed following red clover incorporation in that system suggests that clover residues turned over more quickly than the roots and composted manure which comprised the majority of the C added to the 4YR system (59%, as compared with only 37% of C additions to the 3YR system). Other experiments have shown that composted manure provides long-lasting benefits to soil [53–58], and can build up the labile fraction even in C-saturated soils where total SOC does not accumulate [20]. The extensive root systems associated with "soil-improving" crops such as oats or alfalfa have also been shown to be relatively persistent in soil [13,17], and to contribute disproportionately to SOC and POM-C stocks [16]. Spargo et al. [7] also found that a long alfalfa-based organic rotation that received infrequent manure additions maintained stocks of soil N similar to those of rotations amended more frequently. They attributed this to relatively slow mineralization from alfalfa roots and crowns. Longer periods of dry soil under the LEI systems, particularly the 4YR system, may also have helped reduce decomposition rates. The generally drier soil observed in the surface soils of the LEI systems in spring is consistent with their greater degree of tillage, while the presence of living roots from the oats and legumes in the fall and (in the case of alfalfa) spring also contributed to drier soils, especially at the 10–20 cm depth. Franzluebbers and Arshad [55] found soil drying by roots contributed to greater POM-C pools in subsurface depths. This effect would be stronger for the 4YR system, which included two years of these longer-season crops.

The lack of response of BD or %WSA to either forage crop roots or plowing is consistent with observations by Yoo and Wander [59] made on a similar silty clay loam soil where aggregate turnover was found to be slow and SQIs were little altered by tillage. However, the slight tendency for %WSA to be more stratified in the 3YR system (relatively low in the surface 10 cm in comparison with the 10–20 cm depth) hints that the higher tillage frequency in that system may eventually become detrimental to surface soil structure. Reduced tillage frequency in the 4YR system may also contribute to this system’s ability to maintain similar stocks of organic materials; this and other works [26] indicate substantial SOC gains may be made by even a slight reduction in tillage frequency. The overall similarity in the performance of the 3YR and 4YR rotations suggests that input intensity in the former could be reduced without decreasing yield or soil quality. Spargo et al. [7] have previously noted that animal manure applications can be reduced in established systems when legume cover crops are also used.

### 4.3 Plant responses to soil quality changes

The notion that the maize RLD would reflect soil quality characteristics was supported by the greater stratification of roots that was observed in the 2YR system, that echoed the observed stratification of biochemical SQIs. It is unlikely that root stratification occurred in response to physical limitations in the 2YR system given the low BD and high %WSA found in that system [60], the fact that %WFPS was always lower than the 60% threshold at which oxygen is expected to become limiting [36], and the absence of any significant relationship between average maize root diameter and soil BD [32]. Differences in resource richness seem to better explain patterns of maize root distribution. Roots proliferate in areas of greater nutrient concentration [31,61] and a relatively dry surface soil encourages deeper maize root growth [33], which could help explain why maize roots in the LEI systems were evenly arrayed through the top 20 cm of soil. In contrast, the spring surface soil of the 2YR system provides a water- and nutrient-rich environment that gives little incentive for young roots to explore the deeper soil. The more uniformly distributed LEI maize roots may have been able to exploit more of the soil's water and available N [62,63], to gain improvements in maize N-use efficiency observed in those systems [2]. Concentration of roots in a shallower depth in the 2YR system may also be a disadvantage as this can result in localized depletions in water or nutrients and provide conditions conducive to the spread of disease [11].

The idea that maize yield is positively related to resource richness that is evinced by labile organic matter (POM-C and PMN) is supported by the findings of Spargo et al. [7], who found a strong linear relationship between maize yields and soil PMN in a 13-year trial of organic and conventional systems under different tillage regimes. Nyiraneza et al. [58] also attributed increased maize yields observed in a long-term manure experiment in Canada to measured increases in PMN and available NO_3_-N.

While stratification differences were not significant in soybean RLD, marginally significant differences among soybean root C to N ratios suggest that LEI soybeans also benefited from improved N nutrition. This idea is supported by the significantly larger soybean biomass observed in both LEI systems. Enhanced subsurface N abundance may be an advantage to soybeans. While mineral N in the surface soil is known to reduce biological N-fixation, mineral N placed at about 20 cm is below the nodulation zone and so reduces fixation less, and leads to increased N uptake and yields [64,65]. This expansion of the rhizosphere helps explain why soybean yields and NUE tend to be greater in the LEI than 2YR systems.

Changes in resource distribution reflected by the pattern and abundance of SQIs are likely to have economic implications. A recent economic analysis of the study that was based on 2003–2010 data found that over its history, the net returns to land and management were similar in all systems [66] despite the fact that the LEI systems reduce the proportion of years under grains and increase labor requirements. The LEI systems performed as well as the conventional system due to their greater grain yields and reduced fossil energy and chemical costs.

## Conclusions

This experiment sought to identify links between specific components of LEI management (i.e. inclusion of legumes or small grains, manure incorporation, etc.) and soil quality and root response within an established cropping systems experiment to better understand how LEI systems achieve equal or greater grain yields with lower external inputs than their conventionally managed counterpart. Our results suggest that in Marsden’s fine-textured, well-structured soils, deep incorporation of C and N through tillage in the LEI systems is a main driver of their increased efficiencies. These findings may not be applicable to soils that are more susceptible to erosion or structural degradation by tillage.

Soil quality changes did not reflect the quantity of organic material added in these soils. Because SOC, POM-C and PMN stocks did not increase with C additions, we suspect that these soils have reached saturation or are poised at an equilibrium where they are no longer accumulating C. Mechanisms such as priming, material recalcitrance, and differences in soil moisture that alter decay dynamics within these three systems appear to interact to produce similar dynamic C equilibrium levels. Even though total SOC stock size is similar in all systems, soil quality differences are agronomically significant. Our results suggest that maize and soybeans in the LEI systems are benefiting from the existence of a large pool of labile material present at the 10–20 cm soil depth. This results in somewhat higher stocks of labile material in the LEI systems. Resource distribution is reflected by root activity. Maize roots in the conventional system are concentrated in the top 10 cm of soil, where high spring levels of water and nutrients gave no incentive for roots to explore deeper. In contrast, the LEI maize roots, with their relatively dry surface soil in spring and large stocks of labile materials in the 10–20 cm depth, grew uniformly through the top 20 cm. Maize roots growing in LEI systems were able to exploit a larger volume of soil and thus access more water and nutrients during the season. Soybean roots may have benefitted similarly by accessing soil N below the nodulation zone. This study of in situ measurements of three complex systems suggests that resource distribution, as well as abundance, is an important component of soil health that can be linked to system productivity and efficiency.

## Supporting Information

S1 Dataset Raw data for all soil and root parameters, Fall 2008-Spring 2010. (CSV)

S2 Dataset Raw data for carbon inputs to all crop phases. (CSV)

S1 Table Analysis of variance on means and stratification ratios on soils collected in spring, summer and fall of 2009. (XLSX)

S2 Table Soil parameter means for soils collected in spring, summer and fall of 2009 for all crop phases. (XLSX)

S3 Table Parameter estimates of linear regressions between summer root parameters and bulk density (Pr>F in parenthesis). (XLSX)
